# Genome-Resolved Meta-Analysis of the Microbiome in Oil Reservoirs Worldwide

**DOI:** 10.3390/microorganisms9091812

**Published:** 2021-08-26

**Authors:** Kelly J. Hidalgo, Isabel N. Sierra-Garcia, German Zafra, Valéria M. de Oliveira

**Affiliations:** 1 Microbial Resources Division, Research Center for Chemistry, Biology and Agriculture (CPQBA), University of Campinas–UNICAMP, Av. Alexandre Cazellato 999, 13148-218 Paulínia, Brazil; vmaia@cpqba.unicamp.br; 2 Graduate Program in Genetics and Molecular Biology, Institute of Biology, University of Campinas (UNICAMP), Rua Monteiro Lobato 255, Cidade Universitária, 13083-862 Campinas, Brazil; 3 Biology Department & CESAM, University of Aveiro, Aveiro, Portugal, Campus de Santiago, Avenida João Jacinto de Magalhães, 3810-193 Aveiro, Portugal; inatalia.sierra@gmail.com; 4 Grupo de Investigación en Bioquímica y Microbiología (GIBIM), Escuela de Microbiología, Universidad Industrial de Santander, Cra 27 calle 9, 680002 Bucaramanga, Colombia; geralzaf@uis.edu.co

**Keywords:** petroleum, metagenomics, core microbiota, functional potential, oil fields

## Abstract

Microorganisms inhabiting subsurface petroleum reservoirs are key players in biochemical transformations. The interactions of microbial communities in these environments are highly complex and still poorly understood. This work aimed to assess publicly available metagenomes from oil reservoirs and implement a robust pipeline of genome-resolved metagenomics to decipher metabolic and taxonomic profiles of petroleum reservoirs worldwide. Analysis of 301.2 Gb of metagenomic information derived from heavily flooded petroleum reservoirs in China and Alaska to non-flooded petroleum reservoirs in Brazil enabled us to reconstruct 148 metagenome-assembled genomes (MAGs) of high and medium quality. At the phylum level, 74% of MAGs belonged to bacteria and 26% to archaea. The profiles of these MAGs were related to the physicochemical parameters and recovery management applied. The analysis of the potential functional core in the reservoirs showed that the microbiota was specialized for each site, with 31.7% of the total KEGG orthologies annotated as functions (1690 genes) common to all oil fields, while 18% of the functions were site-specific, i.e., present only in one of the oil fields. The oil reservoirs with a lower level of intervention were the most similar to the potential functional core, while the oil fields with a long history of water injection had greater variation in functional profile. These results show how key microorganisms and their functions respond to the distinct physicochemical parameters and interventions of the oil field operations such as water injection and expand the knowledge of biogeochemical transformations in these ecosystems.

## 1. Introduction

Oil reservoirs are unique subsurface environments often located deep in the earth, characterized by a complex matrix of hydrocarbons, low oxygen levels, and a long history of separation from the surface [1]. Currently, while a plethora of bacteria and archaea have been detected in petroleum reservoirs, some patterns in microbial taxonomic composition have been identified that correlate to specific environmental factors such as temperature and depth, with little role for the geographic distance between petroleum reservoirs worldwide [2,3]. Shallow and low-temperature reservoirs show an abundance of Epsilonbacteria and Deltaproteobacteria members, while deeper high-temperature reservoirs are enriched in Clostridiales and Thermotogales. Despite these specific taxa that distinguish oil reservoirs according to their attributes, a core microbiome composition across the world has been identified, showing that the bacterial classes Gammaproteobacteria, Clostridia, and Bacteroidia and the archaeal class Methanomicrobia are ubiquitous in petroleum reservoirs [4].

There have been several studies using metagenomics aiming to provide insights into the functional potential of microorganisms in oil field environments [5,6,7]. Pioneering studies comparing metagenomes from oil reservoirs with metagenomes from hydrocarbon-rich or non-hydrocarbon-rich environments reported higher abundances of genes related to anaerobic hydrocarbon metabolism and methanogenesis in samples from oil reservoirs [5,8]. However, the metabolic capability of individual organisms and intra-community interactions remains largely unresolved due to a lack of enough genome information of biochemical transformations in oil reservoirs. Emerging approaches to recover MAGs from metagenomic data [8,9,10] allow the recovery of microbial genomes and access the functional potential of keystone taxa, including the uncultivated ones, and their interactions.

At present, several metagenomic datasets derived from oil reservoirs located in China [2,11,12], Denmark [13], Alaska [6], and Brazil [3] have been added to public repositories. Most of these metagenomic sequence data were analyzed as isolated niche studies, however, no analysis has been carried out so far on the universal patterns or core functions of microorganisms in such deep environments. The understanding of the functional role and specificity of microorganisms inhabiting the subsurface biosphere requires the integration of the metagenomic data using novel tools. 

Here, we aimed to integrate the publicly available metagenomic data from oil reservoirs to deeply characterize community composition and metabolic profiles in petroleum reservoirs worldwide. The questions that guided our work were: (*i) Is there a potential functional core shared by microorganisms in oil reservoirs worldwide? (ii) Are there metabolic processes that are specific to particular petroleum reservoirs? (iii) Are pathways for hydrocarbon degradation in oil reservoirs different from the ones in other environments?* The answers to these questions are important for the comprehension of metabolic capabilities, biogeochemical cycles, and geological resources in deep subsurface environments.

## 2. Material and Methods

### 2.1. Data Acquisition 

Seventeen datasets derived from publicly available shotgun metagenomes associated with oil fields were downloaded and used in this study. These data correspond to 17 samples distributed in six studies [1,2,3,6,11,12]. Metagenome data from oil reservoirs with different recovery management and temperatures located in different regions were included to investigate the influence of geographic location, temperature, and anthropogenic interventions on functional and taxonomic profiles of the indigenous microbial communities through the recovery of petroleum-associated MAGs. All sequence data were obtained from the National Center for Biotechnology Information (NCBI). The sample accession number, location, type of sample, and related references are listed in Supplementary Table S1.

#### Description of Datasets

**Figure 1:**
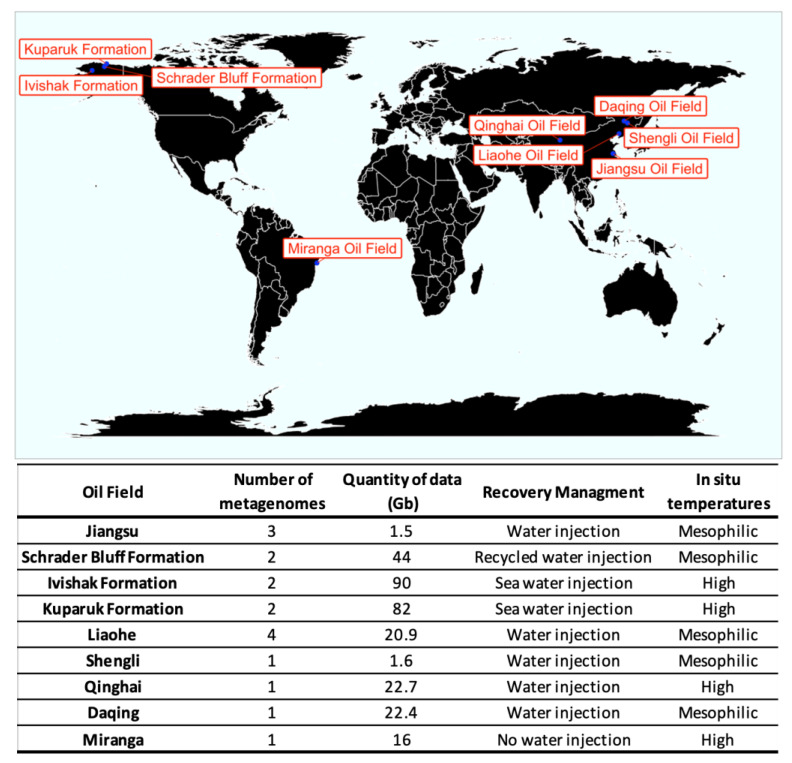
Geographic distribution of metagenomes. The number of metagenomes and the amount of information in gigabytes from each site are in parenthesis.

The geographic distribution of the metagenomes analyzed and the amount of data in Gigabytes from each site were plot in Figure 1. Five oil fields from China comprising ten metagenomes and almost 69 Gb of information were analyzed [1,2,11,12]. Three formations in Alaska with 6 metagenomes and 216 Gb [6] were analyzed. Finally, one metagenome with 16 Gb of data from a Brazilian oil field was also included in the meta-analysis [3]. 

**Figure 2:**
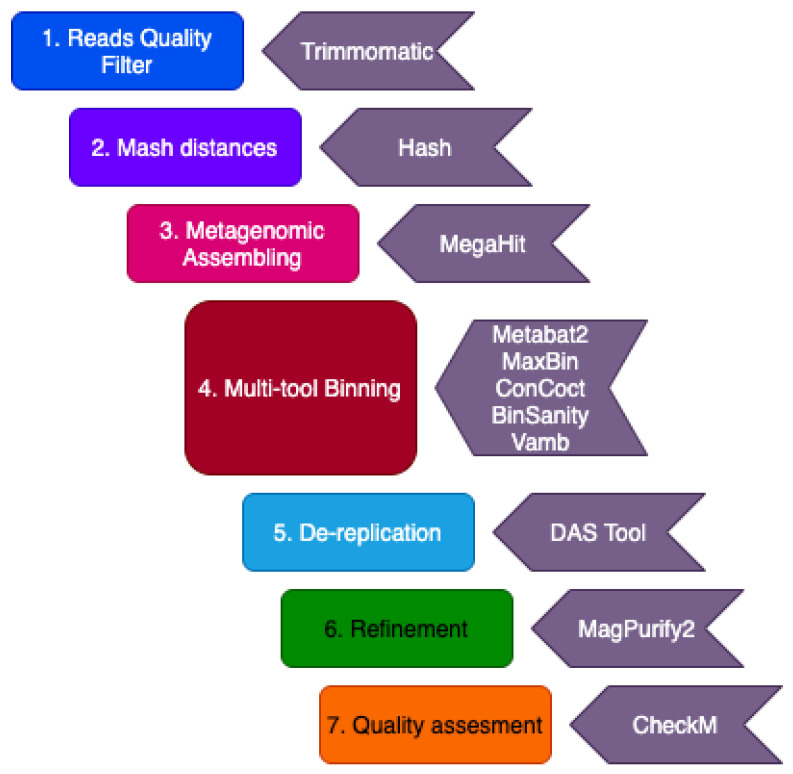
Computational pipeline for obtaining MAGs.

All downloaded metagenome datasets were submitted to the binning pipeline approach established in this work (Figure 2) to recover MAGs. The study performed by Liu et al. [11] described the metabolic potential and activity of the microbial communities obtained from the Jiangsu Oil Reservoir (China). They sampled produced water from three different wells, named W2-71, W9-18, and W15-5, and sequenced in Illumina MiSeq platform (2 × 75 bp). Hu et al. [6] used metagenomic shotgun sequencing of six samples from two North Slope oil fields in Alaska to compare the microbial community across the range of physical and chemical conditions and to predict metabolic roles. The produced water samples were obtained from different depths and temperatures, with or without hydrogen sulfide production (souring), and with or without impact by seawater injection. Samples SB1 and SB2 were from Schrader Bluff formation, with a temperature range of 24–27 °C and depths from 1200 to 1400 m. Samples K2 and K3 were from the Kuparuk formation, with a temperature range between 47 and 70 °C and depths between 1785 and 2150 m. And finally, samples I1 and I2 were collected from the Ivishak Formation, the hottest and deepest reservoir of the study, with a temperature range between 80 and 83 °C and depths of 2750 to 3100 m. In the study of Wang et al. [1], the community structure and metabolic potential of oil-water mixture samples collected from the water-flooded Shen84 oil reservoir (Liaohe Oil Field, in China) was investigated. Wells 6111_O and 6111_W showed medium permeability, medium oil saturation, and active edge water, well AJ-5 showed high permeability, high soil saturation, and intensive anthropogenic perturbation, and well 71551 showed extremely low permeability and low oil saturation. Song et al. [12] studied a water-flooded petroleum reservoir at the Shengli Oil Field, located in China. They sampled produced fluids from the wellhead of the production well to assess the taxonomic and functional information of the subsurface microbial community. In the study performed by Nie et al. [2], crude oil metagenomes from two oil reservoirs in Daqing and Qinghai Oil Fields in China were analyzed. The oil samples were collected from the wellhead of the high-temperature Qinghai (QH) Oil Field and the wellhead of the mesophilic Daqing Oil Field (DQ), to compare the microbiome taxonomic and functional profiles. Finally, Sierra-Garcia et al. [3] sequenced the metagenome from a crude oil sample collected from a non-water flooded and high-temperature Brazilian oil reservoir located at Miranga Oil Field in Brazil (BA-1) to assess the functional genetic potential of the microbial community. Due to the differences in the stage of recovery management and temperatures among the wells, five groups of samples were defined: (i) without water injection and high temperature; (ii) with water injection and high temperature; (iii) seawater injection and high temperature; (iv) water injection and mesophilic temperatures; (v) recycled water injection.

### 2.2. Computational Analysis

#### 2.2.1. Quality Control and Filtering

In this study, we used metagenomic sequencing datasets previously obtained from oil reservoirs worldwide to recover MAGs. Sequence datasets were downloaded from databases and processed according to Figure 2. Briefly, quality control of sequencing reads was performed using FASTQ v.0.11.5 [14]. Adapter presence was detected using BBMap v38.90 [15]. Trimmomatic v. 0.36 [16] was used to remove the adapters and to trim reads with quality lower than 30 (Phred score) and length smaller than 100 bp. For some metagenomes with low quality and read lengths of 100 bp, Phred scores 20 and 75 bp minimum length were used as the thresholds. 

#### 2.2.2. Metagenome Assembly and Binning

Mash software was used to calculate the pairwise distances between the datasets by employing the MinHash technique [17] (Figure 2), to know which samples could be co-assembled. Metagenome datasets with distance values below 0.1 were co-assembled. Both the trimmed read pairs and the reads left unpaired after quality filtering were used for metagenomic assembly. Assembly and co-assembly modes were performed using Megahit v.1.1.2 [18] with the following parameters: ‘–k-min 27 –k-max 147 –k-step 10 –min-contig-len 500’. The quality of the generated assemblies was assessed using MetaQUAST v5.0.2 [19]. The coverage profile of the contigs was obtained by using Bowtie2 v2.3.5.1 [20] read alignment program to map the reads of each sample to each assembly. SAM files generated were translated to BAM files, then sorted and indexed using SAMtools v1.9 [21].

Oil reservoir-associated MAGs were generated per assembly using five different binning tools (Figure 2) with default options: MetaBAT2 v2.15 [22], MaxBin v2.2.7 [23], CONCOCT v1.1.0 [24], BinSanity v.0.5.3 [25], and Vamb v.3.0.2 [26], which use a combination of sequence composition and coverage information from the coverage profile. DAS Tool v1.1.2 [27] with option ‘*--score_threshold 0.0*’ was used to combine MAGs produced by the five tools from each assembly to generate a non-redundant MAGs set. The resulting bins were analyzed for quality, contamination, and completeness using CheckM v1.1.3 [28], which also uses HMMER v.3.2.1 [29] as a third-party software to compare the obtained bin against the single-copy gene profile of the genome of the affiliated taxon and to quantify how many genes are in the bin (completeness) and how many are in single-copy (contamination). Magpurify2 (https://github.com/apcamargo/MAGpurify, accessed on 5 May 2021) was used to refine the obtained MAGs to remove putative contaminant contigs within each MAG (Figure 2). 

MAGs quality was defined according to the MIMAG standard [Completeness–(5*Contamination)] [30]. MAGs with quality scores >50 were used in downstream analyses. High-quality MAGs are defined as completeness >90% and contamination <5% and medium-quality MAGs are defined as completeness >50% and contamination <10%. The mapping rate was estimated using Bowtie2 v1.3.0 [20] to map the reads of each metagenome to the MAGs originated from it; the proportion of reads that mapped with at least 95% identity was obtained with CoverM v0.6.1 (https://github.com/wwood/CoverM, accessed on 5 May 2021) in genome mode.

### 2.3. Taxonomic Assignment and Phylogeny

Taxonomic affiliation of MAGs was carried out using Genome Taxonomy Database and the associated GTDB-Tk toolkit v1.1.0 [31,32,33] and the RefSeq Release 89. GTDB-Tk, which also uses pplacer as a third-party software [34], uses average nucleotide identity (ANI) and genome topology to find the closest genomic relative in its database of >140,000 public prokaryote genomes. A phylogenetic approach was used to place the obtained MAGs in the tree of Bacteria and Archaea. PhylophlAn v3.0 [35] was used to place the genomes in a high-resolution phylogeny, and iTOL [36] was used for the visualization using the Newick file as input. 

### 2.4. Functional Assignment

Genes encoded in MAGs were predicted using Prodigal v.2.6.3 [37] and annotated using sequence aligner Diamond v0.9.30 [38] based on the KEGG database (Kyoto Encyclopedia of Genes and Genomes [39]. After annotation, genes found were filtered based on a list of specific genes related to hydrocarbon metabolism (Supplementary Table S2). The completeness of each pathway was calculated based on the fraction of genes present in KEGG Modules. 

### 2.5. Environment-Specific Orthogroups

Proteins annotated in the oil reservoir MAGs were compared to related organisms to assess functional novelty related to oil reservoir environments. First, MAGs with taxonomic affiliation at least the family level were identified and grouped. Then, protein sequences from species of the same families of the oil reservoir MAGs set were recovered from GTDB (release 89). Only families with at least four genomes were used for this analysis (including the MAGs generated in this study). All-versus-all pairwise comparisons between proteins in each family were performed using Orthofinder v2.5.2 [40], clustering the proteins into orthogroups (orthologous gene cluster), and generating count matrices with the number of genes within each orthogroup per genome. To identify orthogroups that are exclusive to the oil reservoir MAGs (environment-specific orthogroups), tspex v0.6.2 [41] was used to calculate the specificity measure (SPM) of each orthogroup from the matrices. Only orthogroups with at least three genes were used. If any orthogroup was classified as exclusive to the oil reservoir MAGs, the percentage of enrichment in all orthogroups in each family was calculated comparing with the representative genome from the GTDB. The orthogroups with at least 60% of enrichment were selected and functionally annotated using Diamond v. [38] against KEGG [42] and EggNogg [43] databases. Orthogroups with ambiguous functions (different annotations within the orthogroup) were ignored.

### 2.6. Functional Core Analysis

Annotation of a functional core shared among all oil reservoir datasets analyzed was performed based on KEGG databases. Gene annotations from each oil field were pooled and compared to assess both exclusive and common KEGG categories among sites. The percentage of orthologies in each group was calculated. The relative abundance of the functional core among all sites and of the exclusive functions based on Level 2 categories from KEGG orthologies were assessed.

## 3. Results & Discussion

### 3.1. Co-Assembling Statistics

After filtering low-quality reads, metagenomic datasets were processed by grouping metagenomes by read-level similarity (Minhash distances), to determine which of the metagenome sets could be co-assembled. The resultant distance matrix was plotted in a clustering heatmap (Supplementary Figure S1). Datasets with distances below 0.1 were co-assembled. Four co-assemblies and six individual assemblies were performed. In all cases, the co-assemblies corresponded to samples of the same study. Supplementary Table S3 summarizes the statistics of the assemblies.

### 3.2. Binning

The individual assemblies and co-assemblies were binned to MAGs. Merging of bins obtained using the pipeline described herein (Figure 2) yielded 176 MAGs. Quality evaluation results indicated that 74 of MAGs had estimated completeness higher than 50% and contamination less than 10% and were classified as medium quality, while 74 MAGs had completeness higher than 90% and contamination less than 5% and were classified as high-quality MAG, according to the MiMAG standard [30] (Supplementary Figure S2). MAGs with low-quality were not used for further analyses.

### 3.3. Taxonomic Affiliation and Phylogeny

**Figure 3:**
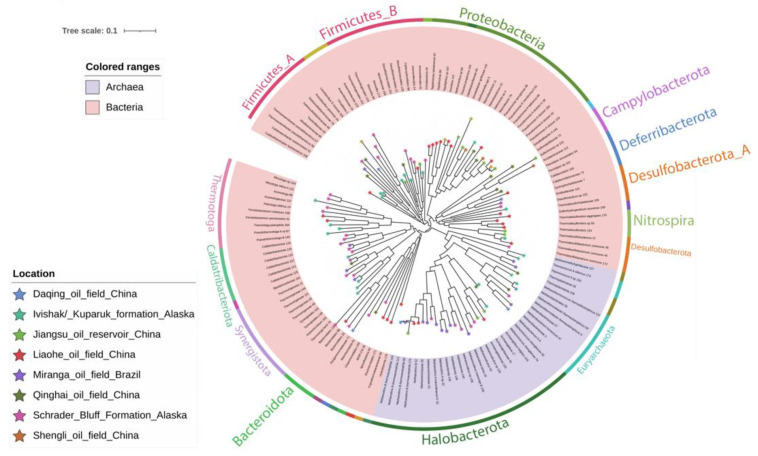
Phylogenetic tree of the 148 MAGs recovered from the oil reservoirs. The tree is decorated with colored backgrounds corresponding to domains, the most represented phyla are labeled in color in the outermost ring. The star shapes in the tips of the branches indicate the location of origin of the MAGs (see legend).

For the taxonomic assignment of MAGs, a phylogenetic tree was reconstructed using the 148 MAGs obtained in this study (Figure 3). One hundred and ten MAGs belonged to the Bacteria domain while 38 belonged to Archaea. The MAGs were grouped into 25 bacterial phyla and four archaeal phyla. The tree showed three large clusters representing the phyla Firmicutes (21 MAGs), Proteobacteria (19 MAGs), and Halobacterota (24 MAGs) (Figure 3). Smaller clades represented the Campylobacterota, Deferribacterota, Nitrospira, Desulfobacterota, Thermotoga, Euryarchaeota, among others. The most representative family was *Methanotrichaceae* with nine MAGs, followed by *Pseudomonadaceae* with seven MAGs, *Thermovirgaceae* with six MAGs, and *Archaeoglobaceae* with four MAGs. At the genus level, MAGs were assigned to 94 different genera. The most represented ones were the methanogenic archaeon *Methanothrix*, with five MAGs, and the proteobacterium *Pseudomonas*, also with five MAGs, followed by *Thermodesulfobacterium* (4) and *Thermodesulfovibrio* (3). The taxonomic assignment of all MAGs is detailed in Supplementary Table S4.

The first group of samples (without water injection plus high temperature) was composed only by BA-1 sample from Miranga Oil Field, the solely well that had not been water-flooded among the samples studied here. This well may reflect, to some degree, the indigenous thermophilic microbial communities in oil reservoirs before flooding operations. Sixteen MAGs were recovered from this sample, of which 11 MAGs were distributed in three archaeal phyla and five MAGs in four bacterial phyla (Figure 3). The Archaeal phylum Halobacterota was represented by 50% of the MAGs. The bacterial MAGs were assigned to the phyla Synergistota, Firmicutes, Patescibacteria, and Caldatribacteriota. At the genus level, 13 out of 16 MAGs could not be assigned to any taxa, suggesting that the reservoir harbors several unknown taxa. Two MAGs, including one affiliated to the Microgenomatia class and another affiliated to the *Tissierellaceae* family were found exclusively in this reservoir. The class Microgenomatia belongs to the Patescibacteria, recently proposed as a phylum [33]. The majority of taxa from this phylum have been proposed through the Metagenome-Assembled Genome approach of difficult access environments, such as groundwater, deep-sea sediments and, deep subsurface [44,45,46,47,48,49]. In addition, three of the Microgenomatia genomes were found in activated sludge from a petrochemical plant [50], and one MAG belonging to Patescibacteria was found in crude oil metagenome from an oil field in Wietze, Germany [51]. Microbes from this phylum are characterized by small cell and genome sizes [52], containing only essential genes and lacking many metabolic functions like amino acid or nucleotide biosynthesis [44]. Due to these special characteristics, a parasitic lifestyle has been suggested [8,53]. Two MAGs from the Synergistales order were found in the BA-1 sample from Miranga Oil Field. One of them was affiliated with the *Thermovirgaceae* family. Members of this family were found in a hydrocarbon reservoir that was submitted to a CO_2_ enhanced oil recovery flood 40 years ago [54]. In terms of the archaeal community, this oil reservoir metagenome was characterized by the predominant presence of members of Halobacterota (8) phylum, followed by Thermoplasmota (2) and Euryarchaeota (1). MAGs from Thermoplasmota phylum could not be affiliated to higher taxonomic ranks. MAG2 was affiliated with the *Methanobacter* family. Members of this family are known to reduce H_2_ and CO_2_ to produce methane [55]. In a study across 22 oil reservoirs, Methanobacteria were found in all of them [56]. Zhao et al. [57] analyzed eight oil reservoirs from northern China and found that the core microbiota was composed of three bacterial and eight archaeal genera, including the genus *Methanobacterium* from the *Methanobacteriaceae* family [57]. In this study, one MAG from the family *Methanoregulaceae* was recovered and affiliated to the species *Methanolinea tarda.* Members of *Methanolinea* are hydrogenotrophic archaea [58] and are also found as part of the core in some studies [56,57,59]. Four MAGs were affiliated to the *Methanotrichaceae* family, two of them belonging to *the Methanothrix* genus. Finally, three MAGs were assigned to Halobacterota phylum, without any classification at higher ranks. However, they were placed into the Methanotrichales order clade (Figure 3 and Supplementary Table S4). The family *Methanotrichaceae* was proposed as a new family to replace *Methanosaetaceae*. *Methanothrix* is the only genus in this family, with three species, *M. harundinacea, M. thermoacetophila, and M. soehgenii* [60]. This genus produces methane mainly from acetate, but it is also able to reduce CO_2_ via direct interspecies electron transfer (DIET) with *Geobacter* spp. [61,62]. Members of *Methanothrix* are more frequently dominant in reservoirs from medium to high-temperature reservoirs [56]. 

In the second group of samples (water injection plus high temperature), only the dataset from the Qinghai Oil Field (QH) was included. This oil field has been submitted to water-flooding for 15 years. Binning using this metagenome yielded four archaeal MAGs (Supplementary Table S4), which were assigned to Halobacterota (*Methermicoccus shengliensis*), Euryarchaeota (*Methanothermococcus thermolithotrophicus* and *Methanobacteriaceae* member), and Nanoarchaeota (member of Woesearchaeales order). The last one is an ultra-small hyperthermophilic obligate ectosymbiont with a reduced genome that lives on the surface of several archaeal hosts of the Crenarchaeota phylum [63]. Members of Nanoarchaeota phylum have already been found in hot springs in Yellowstone National Park (United States) under high temperatures (~90 °C) [64]. They are also associated with volcanoes rich in hydrocarbons [64], deep-sea hydrothermal vents, and deep lakes [63,64,65,66,67,68,69,70,71,72,73]. *Methermicoccus shengliensis* is a methylotrophic methanogen. It has been isolated from production water samples from a high-temperature oil reservoir in Japan [74] and from Shengli Oil Field, where the in-situ temperatures range from 75 to 80 °C [75]. *Methermicoccus shengliensis* is a hydrogenotrophic and methylotrophic methanogen [56]. Gao and colleagues investigated the spatial distribution of microbial communities and their drivers in 20 water-flooded oil reservoirs and two that had not been flooded, in China, and observed that *Methanothermococcus* was found more frequently in reservoirs with high salinity [56]. A study in the Xinjiang Luliang long-term water-flooded oil reservoir detected *Methanothermococcus thermolithotrophicus* by *mcrA* sequencing [76]. With regards to the Bacteria Domain, 12 MAGs were recovered from the QH dataset, which belonged to eight phyla, as follows: Campylobacterota (1), Cloacimonadota (1), Deferribacterota (2), Desulfuromonadota (1), Firmicutes (3), Patescibacteria (1), Proteobacteria (2), and Synergistota (1). Two genomes of *Flexistipes* (Deferribacterota phylum) were found. There is evidence that species from this genus, as *F. sinusarabici* (MAG94), have genes that encode for electrically conductive pili (e-pili), similar to *Geobacter* species, that is responsible for the DIET process [77]. *Geoalkalibacter* (MAG146), belonging to Proteobacteria, is a strictly anaerobic bacterium, able to reduce Fe(III). A strain of this genus was isolated from a produced water in the Redwash oil field in Utah, USA, with 52 °C of in situ temperature [78]. This genus was also found in a reservoir that had not been water-flooded [56]. Members of the genera *Pseudomonas, Marinobacter, Sulfurospirillum*, among others, are universally detected in reservoirs with a wide range of temperature, and most of them are hydrocarbon-degraders, surfactant producers, and nitrate or sulfate reducers [56]. Many of these microorganisms are aerobic. Due to the low redox potential in the oil reservoirs, anaerobic and facultative microorganisms are abundant. However, a lot of aerobic microbes are also detected in this environment, and water injection may be closely related to that phenomenon [76].

The third group of samples (seawater injection plus high temperature) were clustered Kuparuk (K2 and K3) and Ivishak formations (I1 and I2). The binning process of these four metagenomes allowed the recovery of 24 MAGs. Some different physicochemical characteristics were found among these samples; first, the temperatures in the Ivishak formation were between 80–83 °C and in the Kuparuk were from 47 to 70 °C, further, the well I2 has the longest history of seawater injection among all samples, and that is reflected by the presence of the *Desulfonauticus* spp. (Desulfobacterota phylum), sulfate-reducing bacteria present in the deep sea [79,80]. On the other hand, the I1 well has only recently been injected with seawater. Only two MAGs were recovered from the I1 sample. One of them was assigned to the thermophilic fermenting anaerobe *Thermoanaerobacter pseudethanolicus* (Firmicutes phylum), which has been isolated from oil reservoirs. This species can respire thiosulfate contributing to souring [81] and, depending on the ecological conditions, may act in syntrophy with acetoclastic methanogens [82]. The second MAG was assigned to *Halomonas*, Gammaproteobacteria class, a known hydrocarbon degrader [83]. MAGs found in the Kuparuk wells were different probably because the temperatures were lower, and the use of seawater injection is more recent than in the other site. These were classified as *Methermicoccus shengliensis* and the hyperthermophilic sulfide producing *Archaeoglobus fulgidus* [59,79], belonging to the Halobacterota phylum, and as the CO_2_/H_2_-reducing methanogen *Methanothermobacter thermautotrophicus* and the sulfide-producing *Thermococcus sibiricus*, members of the Euryarchaeota phylum. The last two are frequently found in oil thermophilic oil reservoirs [54,56,57,76,79]. Seven genomes belonging to Firmicutes phylum were recovered: *Thermoanaerobacter pseudethanolicus* (2), *Caldanaerobacter subterraneus* (1), Thermoacetogeniales (1), Ammonifexales (1), and *Moorellia* (2), which are known to be syntrophic and fermentative bacteria [75,84,85,86]. The family *Thermotogaceae* was represented with three genomes, belonging to two different thermophilic genera, *Thermotoga* and *Pseudothermotoga*. These microbes are indigenous to petroleum reservoirs and more commonly abundant in non-water flooded oil fields [87]. They are fermentative bacteria and have been detected in several high-temperature oil reservoirs [75,81,88,89]. Lastly, one MAG affiliated to *Thermodesulfobacterium commune*, Desulfobacterota phylum, was found. This bacterium can incompletely oxidize fatty acids to produce acetate [90].

The fourth group of samples (water injection plus mesophilic temperatures) were clustered Liaohe, Jiangsu, Shengli, and Daqing Oil Field datasets. In this cluster, 52 MAGs were recovered, of which only ten belonged to the Archaea Domain. As expected, this group of wells was characterized by the presence of many Proteobacteria members, especially the well-known hydrocarbon-degraders and surfactant producers like the facultative anaerobe organotrophs *Pseudomonas* (*P. stutzeri, P. aureginosa, P. balearica*), *Acinetobacter*, *Marinobacterium,* and *Tepidimonas*, and the fermenters and nitrate reducers *Azovibrio* and *Thauera*. In many investigations, these bacteria have been detected in most of the oil reservoirs studied [56] and some authors have proposed these genera as part of the core microbiota of oil reservoirs [57,91,92]. However, they can be more abundant in water-flooded reservoirs [86]. Two MAGs belonging to Firmicutes phylum were recovered, *Lactobacillus ozensis* and *Anoxybacillus gonensis*. species of the fermentative genus *Lactobacillus* have already been detected in oil samples, although its role in oil reservoirs remains unknown [93]. A member of the *Anoxybacillus* genus was isolated from production water of a high-temperature oil reservoir and showed the ability to reduce nitrate and to control sulfate reducers [94]. One MAG assigned to the thermophilic sulfate-reducing genus *Thermodesulfovibrio* (Nitrospira phylum) was reconstructed. Members of this genus were previously detected in oil field environments [95]. Archaeal MAGs were mainly assigned to *Methanotrichaceae* and *Archaeogloboceae* Families and one MAG was assigned to the genus *Methanolobus*, a methylotrophic methanogen belonging to the *Methanosarcinaceae* family [96,97]. This methanogen was predominant in low and medium-temperature oil reservoirs [56].

Finally, in the fifth group of samples (recycled water injection), SB1 and SB2 from the Schrader formation were included. Assembly enabled the recovery of 40 MAGs, of which seven were assigned to the Archaea domain, represented by 5 families, *Methanobacteriaceae, Methanocorpusculaceae, Methanocullaceae, Methanomethylophilaceae,* and *Methanotrichaceae*. The hydrogenotrophic and mesophilic *Methanocalculus* archaeal genus was found in reservoirs without anthropogenic perturbation and were dominant in medium to high-temperature reservoirs [56]. *Methanoculleus* is a methanogen able to reduce CO_2_ in syntrophy with bacteria such as *Syntrophus* and *Marinobacter* [79]. This genus was proposed as part of the core microbiota across several oil reservoirs in China [57] and was found as dominant in low to medium-temperature reservoirs [56]. A study performed by Liang et al. [98] proposed cooperation between *Methanoculleus* and *Anaerolineaceae* members for the n-alkanes degradation. Three MAGs affiliated to the *Anaerolinaceae* family were found in these samples, and other MAGs belonging to the *Kosmotogaceae* family and one to the *Petrotogaceae* family, both from the Thermotogota phylum, were recovered. Members from these two last families have been found in oil field environments [98,99]. Lastly, three MAGs classified as sulfate-reducing bacteria from the Desulfotomaculales order were found*. Desulfotomaculum* has already been proposed as hydrocarbon-degrading bacteria because of high similarity with gene sequences coding for benzyl succinate synthase (*bssA*), responsible for the activation of alkyl-substituted aromatic hydrocarbons [59].

### 3.4. Metabolic Potential 

**Figure 4:**
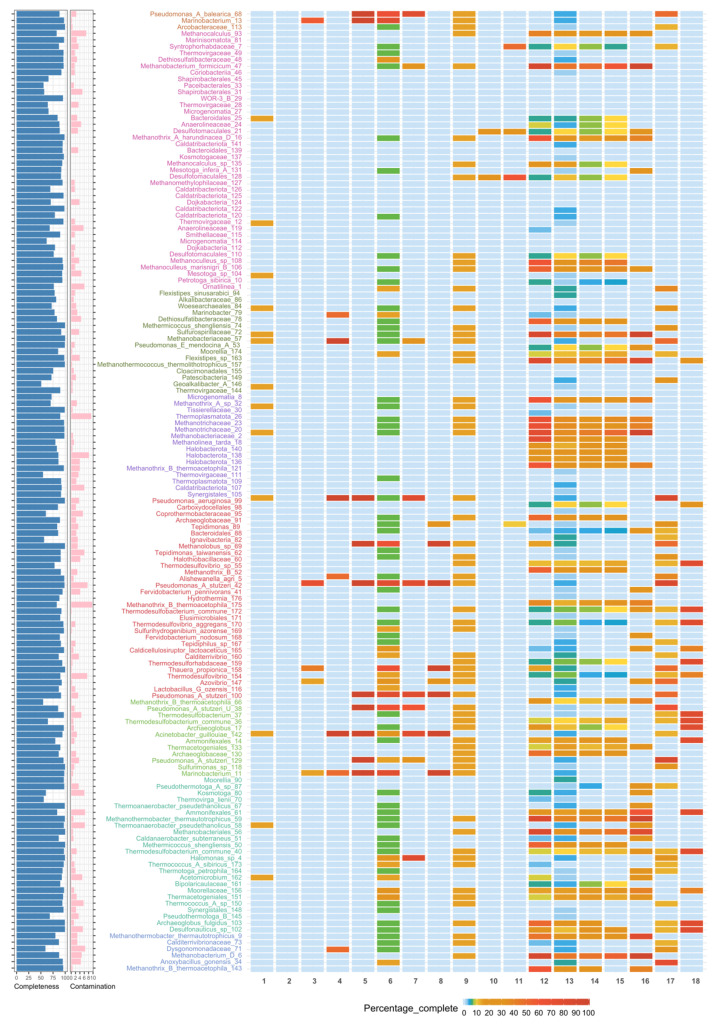
Heat map showing KEGG Module completeness in each MAG. Numbers below the bars: 1—Ring Cleavage via Baeyer–Villiger oxidation; 2—Naphthalene degradation; 3—Biphenyl degradation; 4—Alkane-degradation; 5—Benzoate degradation; 6—Catechol meta-cleavage; 7—Catechol ortho-cleavage; 8—Benzene degradation, aerobic; 9—Phenol degradation, anaerobic; 10—Toluene degradation, anaerobic; 11—Benzoyl-CoA degradation, anaerobic; 12—Methanogenesis, CO_2_; 13—Methanogenesis, acetate; 14—Methanogenesis, methylamines; 15—Methanogenesis, methanol; 16—Coenzyme M biosynthesis; 17—Dissimilatory nitrate reduction; 18—Dissimilatory sulfate reduction. Left bar plots represent completeness (blue bars) and contamination (pink bars). The highest taxonomic affiliation was used. Color taxa names represent the oil field (see key color Figure 3).

Similar to the taxonomic analysis, annotation of the potential metabolism of MAGs revealed the presence of a microbial functional core across all oil fields and the occurrence of exclusive (or site-specific) functions, depending on the recovery management and in situ temperatures (Figure 4, Supplementary Table S2). Without any anthropogenic intervention, the indigenous microbial communities in oil reservoirs are initially dominated by slow-growing anaerobes such as methanogens and some Clostridia members that are adapted to living on a high concentration of hydrocarbons and high temperatures, among other extreme conditions [13]. This was observed in the first group, represented by the sample from the Miranga Oil Field, that was mainly governed by methanogenic archaea, and as consequence, the potential metabolism prevailing was mainly methanogenesis-related (Figure 4, taxa names in purple/modules 12–16). In the second group, which originated from high-temperature water flooded oil reservoir (Figure 4, taxa names in dark green), it was possible to observe diversification in both taxonomic and metabolic levels. In addition to the methanogenesis metabolism (modules 12–16), alkane-degradation (module 4) and dissimilatory nitrate reduction (module 17) were also present. Depending on the water source, the injection can deliver oxidants and different electron acceptors, thus promoting fast-growing microbes as members from Deferribacterota, Proteobacteria, and Bacteroidota phyla, with thermodynamically more favorable metabolism as nitrate or sulfate reduction [13]. In the third cluster, comprising Kuparuk and Ivishak Oil Fields, seawater was mainly used for secondary recovery. The use of seawater can introduce sulfate to the reservoirs, promoting sulfate reduction in the community and the consequent production of H_2_S [100]. *Desulfonauticus* sp. was probably introduced in the oil reservoir by seawater, and the functional analysis of this MAG showed the capability to reduce sulfate with the respective module almost complete (Figure 4, taxa names green/module 18). In this comparative study, the fourth group can be considered as the “greatest level of intervention” amongst all. These were mesophilic water-flooded oil fields. Here, a great shift in the functional profile of the microbial community was observed (Figure 4, taxa names red, light green, and brown). Modules of hydrocarbon aerobic degradation (Figure 4, 3–8) are found in several MAGs. This was expected since samples were dominated by the fast-growing facultative anaerobes and opportunistic members of *Marinobacter*, *Marinobacterium*, and other well-known hydrocarbon degraders as *Pseudomonas* and *Tepidimonas* [3,13,56]. Especially in the Liaohe and Jiangsu oil fields, many genomes with metabolic potential to reduce nitrate and sulfate were recovered. This is in accordance with the fact that recycled treated water was used for secondary recovery in the Schrader Oil Field. From all MAGs obtained in this study, only a few from this group showed metabolic potential to degrade aromatic hydrocarbons anaerobically. These MAGs were associated with Desulfotomaculales and *Syntrophorhabdaceae*. As it was discussed above, members of the *Desulfotomaculum* had already been associated with fumarate activation mechanism in alkyl-substituted aromatic hydrocarbons as toluene and ethylbenzene [59]. The family *Syntrophorhabdaceae* embraces the single mesophilic genus *Syntrophorhabdus*, which is capable of oxidizing phenol p-cresol, 4-hydroxybenzoate, isophthalate, and benzoate in association with an H_2_-scavenging partner (e.g., *Methanospirillum hungatei* or *Desulfovibrio* sp.) [101,102]. Here, MAGs belonging to *Syntrophorhabdaceae* and Desulfotomaculales showed several genes from the module of anaerobic Benzoyl-CoA degradation (module 11), a universal intermediate formed during degradation of aromatic compounds [103]. 

The metabolic analysis carried out in this study also provided evidence that anaerobic hydrocarbon degradation via fumarate addition (module 10) is a rare metabolism detected in oil reservoirs. The latter results are expected once mechanisms alternative to the archetypical fumarate addition reactions (e.g., anaerobic hydrocarbon carboxylation or hydroxylation) are present in oil reservoirs [3,104], which are not described in any KEGG category or module yet and therefore could not be detected in this work. However, genes for further activated hydrocarbon transformations were frequently detected, such as module 9, phenol anaerobic degradation, which was found in 61 MAGs out of 148, suggesting that the organisms represented by these MAGs mediate the downstream degradation of aromatic compounds via anaerobic benzoate degradation. On the other hand, the potential for aerobic hydrocarbon degradation was showed to occur in oil wells submitted to water flooded and in oil reservoirs with mesophilic temperatures. Last but not least, catechol meta-cleavage and all methanogenesis modules were found across all oil fields, suggesting that these metabolisms can be ubiquitous in oil reservoirs.

**Figure 5:**
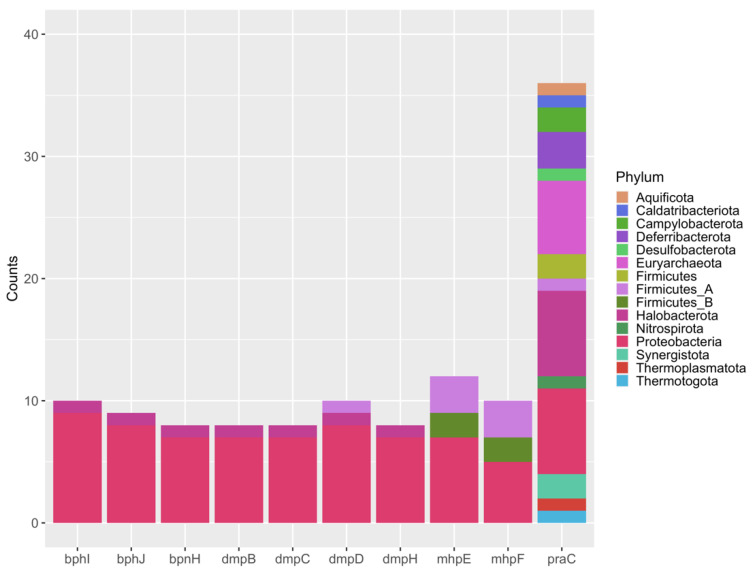
Frequencies of the genes from the Catechol meta-cleavage module across the phyla detected in oils reservoirs under study. Colors represent the phylum annotation.

The module of catechol meta-cleavage degradation was found in 52% of the MAGs (78 MAGs). Even with module completeness of around 10%, it was a relevant result as this metabolic pathway is aerobic and was found in all MAGs belonging to Archaea. A bar plot with the frequency of each gene from catechol meta-cleavage and its phylum affiliation was constructed (Figure 5). The gene *praC*, which encodes the 4-oxalocrotonate tautomerase (4-OT), showed a higher frequency and was present in MAGs affiliated to 14 phyla, mainly in Proteobacteria, Halobacterota and, Euryarchaeota (Figure 5). This gene was found in 15 archaeal MAGs, which were submitted to the PATRIC platform [105] to search for evidence if it is part of an operon. As it can be observed in the MAG comparison, this gene was not found in an operon region (Supplementary Figure S3). In three MAGs, this gene was annotated as a 4-oxalocrotonate tautomerase-like (4-OT) enzyme (MAG17, MAG103, and MAG106) (Supplementary Table S4). In MAG103 (Figure S3a, Supplementary Table S4) and MAG17 (Figure S3b, Supplementary Table S4), assigned to the *Archaeoglobus* genus, the upstream genes found encode unknown proteins, while the downstream genes encode chorismate synthase and Acyl-CoA dehydrogenase. In MAG106 (Figure S3c), assigned to *Methanoculleus marisnigri*, a gene that encodes a haloacid dehalogenase-like hydrolase was found upstream of the *praC* gene. The 4-OT enzyme is part of a group known as promiscuous enzymes since they have multiple low-level catalytic activities in addition to their primary physiological function [106,107]. It is already known that many organisms through Bacteria and Archaea domains harbor 4-OT enzyme homologs which have unidentified physiological functions. Many of these organisms are not aromatic hydrocarbon degraders, suggesting that these homologs might have other functions [106]. 

**Figure 6:**
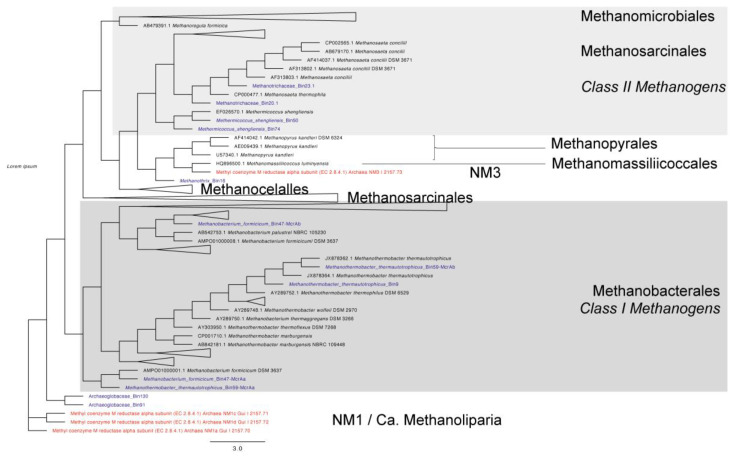
Phylogenetic tree of *mcrA* gene sequences recovered from the archaeal MAGs. Sequences of *mcrA* extracted from MAGs are shown in blue and Ca. *Methanoliparia* (NM1) and NM3 lineages are shown in red. The initial tags for *mcrA* sequences refer to accession numbers.

Several recent studies of the methyl-coenzyme M reductase complex (Mcr) in MAGs have shown that divergent *mcr* -like genes are involved in methane/alkane metabolism. Here, we recovered the *mcrA* genes from MAGs assigned to archaea for further phylogenetic analysis. A phylogenetic tree was constructed using *mcrA* sequences recovered from 11 MAGs and approximately 280 curated *mcrA* sequences recovered from PhyMet^2^ [108]. Interestingly, two MAGs (MAG 47 and MAG 59) (Supplementary Table S4) each contained two different *mcrA* sequences, and two archaeoglobaceae-assigned MAGs contained *mcrA* sequences. In addition to the *mcrA* sequences found, four *mcr*-like sequences belonging to NM1 (*Candidatus Methanoliparia*) and NM3 new lineages were included [109]. According to the phylogenetic analysis (Figure 6), MAG 16 assigned to *Methanothrix* clustered with NM3, Methanopyrales, and Methanomassiliicoccales. NM3 is suggested to perform methyl-dependent hydrogenotrophic methanogenesis with the potential of using methanol and methanethiol [109]. The two MAGs assigned to Archaeoglobacea were related to NM1 lineage. NM1 belongs to Ca. *Methanoliparia* has been reported to be capable of both methane and short-chain alkane metabolisms [109,110]. These results indicate that archaeal MAGs identified in this work could also be involved with hydrocarbon degradation.

### 3.5. Functional Core Analysis

**Figure 7:**
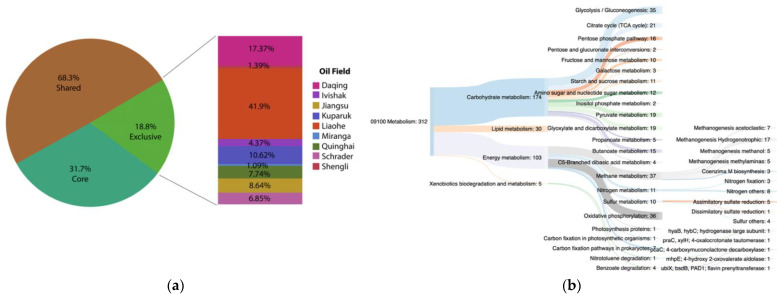

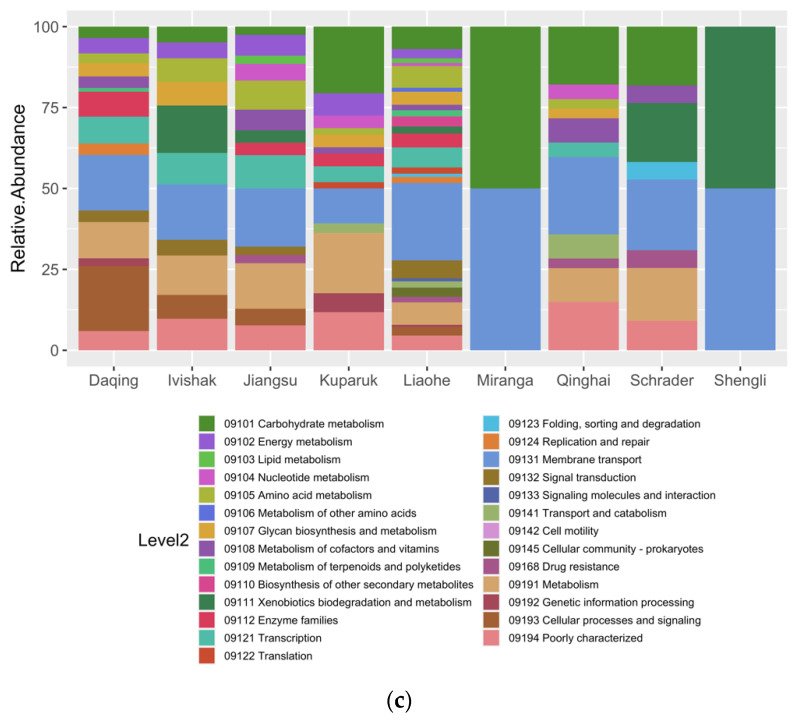
(**a**) Pie chart with percentages of specific KEGG orthologies from each oil field. (**b**) Main core metabolisms. (**c**) Exclusive orthologies at level 2 KEGG categories.

To investigate if there is a potential functional core shared by all oil reservoirs under analysis, KEGG annotations of the MAGs from each oil field were pooled and the KEGG orthologies common to all were assessed. At the same time, the exclusive functions of each oil field were also evaluated. A total of 25,563 ORFs were annotated, distributed in 5332 different KEGG orthologies. Functions that were present in at least 8 out of 9 oil fields were called the potential functional core (1690 genes), which represented 31.7% of total functional annotation (Figure 7a). Site-specific genes (orthologies that were found in one oil field) accounted for 18.8% (1007 KEGG functions) of total annotated KEGG orthologies (Figure 7a). The percentage of specific functions of each site was calculated (Figure 7a). Liaohe Oil Field showed the most distinct metabolic potential compared to the “functional core”, comprising 41.9% of exclusive functions, followed by Daqing Oil Field with 17.37%, and Kuparuk and Jiangsu with 10.62% and 8.64% of exclusive genes, respectively. Shengli and Miranga Oil Fields had the lowest percentage of exclusive functional potential and thus showed the most similar potential metabolism compared to the “core”, with 1.39% and 1.09% of the exclusive KEGG orthologies, respectively. These results suggest a possible correlation between the anthropogenic intervention level and the percentage of exclusive functions. For example, Miranga was the oil field without any intervention, and it had the lower percentage of exclusive functions, while Liaohe with the highest level of perturbations (water injection and mesophilic temperatures) had the highest percentage of exclusive genes (more different from the core).

The main metabolisms encompassed by the potential functional core were assessed (Figure 7b). The functional core comprised almost all metabolic categories. The most abundant core metabolisms were carbohydrate and energy metabolisms, the latter including methane, nitrogen, and sulfur metabolisms. All methanogenesis pathways were found in the functional core, with hydrogenotrophic methanogenesis as the most represented one. In addition, assimilatory sulfate reduction and nitrogen fixation were also part of the functional core. Xenobiotics biodegradation and the metabolism was less represented in the functional core, with five different functions, four from benzoate degradation (genes *praC*, *pcaC*, *mhpE,* and *bsdB*) and one from nitrotoluene degradation (gene *hyaBC*). Genes *mhpE* and *praC* belong to the catechol meta-cleavage pathway, and as was discussed above, gene *praC* encodes to a 4-OT, a promiscuous enzyme. Genes *pcaC* and *bsdB* are also part of aerobic metabolism. These results suggest that aerobic hydrocarbon degradation metabolism is likely an important potential energy strategy for the microbial community in oil fields. However, methanogenesis seems to be the most relevant metabolism in this extreme environment, being omnipresent independently of the good management practice employed. 

Within the exclusive orthologies of each oil field, Ivishak, Schrader, and Shengli Oil Fields showed functional specialization in xenobiotics biodegradation (Figure 7c). For example, in the Shengli Oil Field, more than 50% of specific genes were from the xenobiotics degradation category, and these genes were different from the ones occurring in the other oil fields. These results suggest that in each site microorganisms are specialized in different biodegradation pathways. 

On the other hand, genes for sulfur-oxidizing (*sox* genes) were exclusive in the Liaohe Oil Field. This is consistent with the use of nitrate injection in this reservoir, a promising oil souring control strategy that promotes sulfur oxidation [13,111]. Despite the beneficial role, intermediates from sulfide oxidation are potentially corrosive [112,113,114].

#### Environment-Specific Orthogroups

To explore the functional novelty in the microbiomes of oil reservoirs, a taxonomy-informed approach was used by comparing 67 out of 148 MAGs obtained to related genomes at the family level and identifying orthogroups (cluster of orthologous genes) that are exclusive to the oil reservoir genomes at the family level. Among the 46 families that were analyzed, no unique orthogroups (out of 58,769) were found in the oil field datasets. Due to this result, tspex specificity measure (SPM) output was used to identify enriched orthogroups in the MAGs obtained in this study compared to the representative genomes from GTDB. No orthogroups with at least 60% of enrichment were found (Supplementary Table S5). With the thresholds and conditions of this analysis, no evidence of environment-specific or gene enrichment at the family level was found amongst the microbiomes of oil fields under study, possibly because the families of these environments are already highly adapted to these environments. Maybe at higher taxonomic levels, it would be possible to find exclusive orthogroups related to oil fields.

## 4. Conclusions

Oil reservoirs are very complex niches that can be disturbed by intervention practices for oil recovery (water injection, use of biocides, etc.). Herein, metagenomic information was retrieved from geographical distinct oil reservoirs that corresponded to petroleum fields under different management practices and in situ temperatures. Despite these differences, analysis of metagenomes at the genome level through MAG recovery indicated a core of functions shared by microorganisms in all oil reservoirs under study. The functional core comprised not only basic cellular functions, related to energy and replication, but also methanogenesis pathways and some genes related to aerobic hydrocarbon degradation. On the other hand, hydrocarbon anaerobic degradation via fumarate addition was not relevant. An in-depth analysis of site-specific functions (orthologies that were found only in one oil field) revealed functional specialization in xenobiotics biodegradation in each oil field. Such metabolic specialization is likely driven by the selective pressures imposed by the distinct anthropogenic intervention practices and in situ temperatures. Lastly, the analysis of gene orthologs at the family level did not indicate exclusive or environment-specific genes in petroleum-associated MAGs compared to other environments. Understanding the indigenous and “disturbed” microbial community profiles and their functional potential is essential for the evaluation of the efficiency and consequences of each intervention practice applied to oil reservoirs. Further, knowledge on the taxonomic and metabolic shifts in the oil field microbiome related to anthropogenic intervention is important to design future rationale and efficient oil recovery and toxic pollutant mitigation measures.

## Supplementary Materials

The following are available online at https://www.mdpi.com/article/10.3390/microorganisms9091812/s1, Figure S1: Pairwise distances between the metagenomic datasets. Figure S2: Quality characteristics across low (*n* = 28), medium (*n* = 74) and high quality (*n* = 74) MAGs. (a) Distribution across completeness, (b) Distribution across contamination, (c) Quality dispersion, yellow dot line indicates the cut-off for medium-quality MAGs; green dot line indicates the cut-off for high-quality MAGs. The colors of the dot represent the oil reservoir and respective geographic location where the MAGs come from. MAGs at right and above the yellow and green dot lines were used for the downstream analyses. Figure S3: Schematic representation of the sequence annotation of the 10 Kb around the *praC* gene in three MAGs obtained in this study in comparison with closely related public genomes. (a) MAG17, (b) MAG103, (c) MAG106. Red arrows represent the *praC* gene. Table S1: Metagenome datasets information. Table S2: List of specific genes according to KEGG database. Table S3: Assemblies statistics. Table S4: Complete list of MAGs obtained. Table S5: Edited Tspex comparison matrix. It was calculated the differences between SPM from oil fields’ MAGs and from families of GTDB. Negative values mean no enrichment. Positive values mean enrichment of the orthogroups in each family from oil field MAGs compared with families genomes from the GTDB. Value 1 completely enrichment orthogroup.

## References

[1] Wang X., Li X., Yu L., Huang L., Xiu J., Lin W., Zhang Y. (2018). Characterizing the microbiome in petroleum reservoir flooded by different water sources. Sci. Total Environ..

[2] Nie Y., Zhao J.-Y., Tang Y.-Q., Guo P., Yang Y., Wu X.-L., Zhao F. (2016). Species divergence vs. functional convergence characterizes crude oil microbial community assembly. Front. Microbiol..

[3] Sierra-Garcia I.N., Belgini D.R.B., Torres-Ballesteros A., Paez-Espino D., Capilla R., Santos Neto E.V., Gray N., de Oliveira V.M. (2020). In depth metagenomic analysis in contrasting oil wells reveals syntrophic bacterial and archaeal associations for oil biodegradation in petroleum reservoirs. Sci. Total Environ..

[4] Sierra-Garcia I.N., Dellagnezze B.M., Santos V.P., Capilla R., Santos Neto E.V.S., Gray N., Oliveira V.M. (2017). Microbial diversity in degraded and non-degraded petroleum samples and comparison across oil reservoirs at local and global scales. Extremophiles.

[5] An D., Caffrey S.M., Soh J., Agrawal A., Brown D., Budwill K., Dong X., Dunfield P.F., Foght J., Gieg L.M. (2013). Metagenomics of hydrocarbon resource environments indicates aerobic taxa and genes to be unexpectedly common. Environ. Sci. Technol..

[6] Hu P., Tom L., Singh A., Thomas B.C., Baker B.J., Piceno Y.M., Andersen G.L., Banfield J.F. (2016). Genome-resolved metagenomic analysis reveals roles for candidate phyla and other microbial community members in biogeochemical transformations in oil reservoirs. mBio.

[7] Kotlar H.K., Lewin A., Johansen J., Throne-Holst M., Haverkamp T., Markussen S., Winnberg A., Ringrose P., Aakvik T., Ryeng E. (2011). High coverage sequencing of DNA from microorganisms living in an oil reservoir 2.5 kilometres subsurface. Environ. Microbiol. Rep..

[8] Hug L.A., Baker B.J., Anantharaman K., Brown C.T., Probst A.J., Castelle C.J., Butterfield C.N., Hernsdorf A.W., Amano Y., Ise K. (2016). A new view of the tree of life. Nat. Microbiol..

[9] Parks D.H., Rinke C., Chuvochina M., Chaumeil P.-A., Woodcroft B.J., Evans P.N., Hugenholtz P., Tyson G.W. (2017). Recovery of nearly 8,000 metagenome-assembled genomes substantially expands the tree of life. Nat. Microbiol..

[10] Tyson G.W., Chapman J., Hugenholtz P., Allen E.E., Ram R.J., Richardson P.M., Solovyev V.V., Rubin E.M., Rokhsar D.S., Banfield J.F. (2004). Community structure and metabolism through reconstruction of microbial genomes from the environment. Nature.

[11] Liu Y.-F., Galzerani D.D., Mbadinga S.M., Zaramela L.S., Gu J.-D., Mu B.-Z., Zengler K. (2018). Metabolic capability and in situ activity of microorganisms in an oil reservoir. Microbiome.

[12] Song Z., Chen S., Zhao F., Zhu W. (2019). Whole metagenome of injected and produced fluids reveal the heterogenetic characteristics of the microbial community in a water-flooded oil reservoir. J. Pet. Sci. Eng..

[13] Vigneron A., Alsop E.B., Lomans B.P., Kyrpides N.C., Head I.M., Tsesmetzis N. (2017). Succession in the petroleum reservoir microbiome through an oil field production lifecycle. ISME J..

[14] Andrews S. (2010). FastQC: A Quality Control Tool for High Throughput Sequence Data. https://www.bioinformatics.babraham.ac.uk/projects/fastqc/.

[15] Bushnell B., Rood J., Singer E. (2017). BBMerge—Accurate paired shotgun read merging via overlap. PLoS ONE.

[16] Bolger A.M., Lohse M., Usadel B. (2014). Trimmomatic: A flexible trimmer for Illumina sequence data. Bioinformatics.

[17] Ondov B.D., Treangen T.J., Melsted P., Mallonee A.B., Bergman N.H., Koren S., Phillippy A.M. (2016). Mash: Fast genome and metagenome distance estimation using MinHash. Genome Biol..

[18] Li D., Liu C.-M., Luo R., Sadakane K., Lam T.-W. (2015). MEGAHIT: An ultra-fast single-node solution for large and complex metagenomics assembly via succinct de Bruijn graph. Bioinformatics.

[19] Mikheenko A., Saveliev V., Gurevich A. (2016). MetaQUAST: Evaluation of metagenome assemblies. Bioinformatics.

[20] Langmead B., Salzberg S.L. (2012). Fast gapped-read alignment with Bowtie 2. Nat. Methods.

[21] Li H., Handsaker B., Wysoker A., Fennell T., Ruan J., Homer N., Marth G., Abecasis G., Durbin R. (2009). The sequence alignment/map format and SAMtools. Bioinformatics.

[22] Kang D.D., Froula J., Egan R., Wang Z. (2015). MetaBAT, an efficient tool for accurately reconstructing single genomes from complex microbial communities. PeerJ.

[23] Wu Y.-W., Simmons B.A., Singer S.W. (2015). MaxBin 2.0: An automated binning algorithm to recover genomes from multiple metagenomic datasets. Bioinformatics.

[24] Alneberg J., Bjarnason B.S., de Bruijn I., Schirmer M., Quick J., Ijaz U.Z., Lahti L., Loman N.J., Andersson A.F., Quince C. (2014). Binning metagenomic contigs by coverage and composition. Nat. Methods.

[25] Graham E.D., Heidelberg J.F., Tully B.J. (2017). BinSanity: Unsupervised clustering of environmental microbial assemblies using coverage and affinity propagation. PeerJ.

[26] Nissen J.N., Johansen J., Allesøe R.L., Sønderby C.K., Armenteros J.J.A., Grønbech C.H., Jensen L.J., Nielsen H.B., Petersen T.N., Winther O. (2021). Improved metagenome binning and assembly using deep variational autoencoders. Nat. Biotechnol..

[27] Sieber C.M.K., Probst A.J., Sharrar A., Thomas B.C., Hess M., Tringe S.G., Banfield J.F. (2018). Recovery of genomes from metagenomes via a dereplication, aggregation and scoring strategy. Nat. Microbiol..

[28] Parks D.H., Imelfort M., Skennerton C.T., Hugenholtz P., Tyson G.W. (2015). CheckM: Assessing the quality of microbial genomes recovered from isolates, single cells, and metagenomes. Genome Res..

[29] Wheeler T.J., Eddy S.R. (2013). nhmmer: DNA homology search with profile HMMs. Bioinformatics.

[30] Bowers R.M., Kyrpides N.C., Stepanauskas R., Harmon-Smith M., Doud D., Reddy T.B.K., Schulz F., Jarett J., Rivers A.R., Eloe-Fadrosh E.A. (2017). Minimum information about a single amplified genome (MISAG) and a metagenome-assembled genome (MIMAG) of bacteria and archaea. Nat. Biotechnol..

[31] Chaumeil P.-A., Mussig A.J., Hugenholtz P., Parks D.H. (2019). GTDB-Tk: A toolkit to classify genomes with the genome taxonomy database. Bioinformatics.

[32] Parks D.H., Chuvochina M., Chaumeil P.-A., Rinke C., Mussig A.J., Hugenholtz P. (2020). A complete domain-to-species taxonomy for Bacteria and Archaea. Nat. Biotechnol..

[33] Parks D.H., Chuvochina M., Waite D.W., Rinke C., Skarshewski A., Chaumeil P.-A., Hugenholtz P. (2018). A standardized bacterial taxonomy based on genome phylogeny substantially revises the tree of life. Nat. Biotechnol..

[34] Matsen F.A., Kodner R.B., Armbrust E.V. (2010). Pplacer: Linear time maximum-likelihood and Bayesian phylogenetic placement of sequences onto a fixed reference tree. BMC Bioinform..

[35] Segata N., Börnigen D., Morgan X.C., Huttenhower C. (2013). PhyloPhlAn is a new method for improved phylogenetic and taxonomic placement of microbes. Nat. Commun..

[36] Letunic I., Bork P. (2019). Interactive tree of life (iTOL) v4: Recent updates and new developments. Nucleic Acids Res..

[37] Hyatt D., Chen G.-L., LoCascio P.F., Land M.L., Larimer F.W., Hauser L.J. (2010). Prodigal: Prokaryotic gene recognition and translation initiation site identification. BMC Bioinform..

[38] Buchfink B., Xie C., Huson D.H. (2014). Fast and sensitive protein alignment using DIAMOND. Nat. Methods.

[39] Kanehisa M., Goto S. (2000). KEGG: Kyoto encyclopedia of genes and genomes. Nucleic Acids Res..

[40] Emms D.M., Kelly S. (2019). OrthoFinder: Phylogenetic orthology inference for comparative genomics. Genome Biol..

[41] Camargo A.P., Vasconcelos A.A., Fiamenghi M.B., Pereira G.A., Carazzolle M.F. (2020). Tspex: A Tissue-Specificity Calculator for Gene Expression Data. https://assets.researchsquare.com/files/rs-51998/v1/ec31ccc0-329a-46e0-bfef-c9e409b1edcb.pdf?c=1596577796.

[42] Kanehisa M., Furumichi M., Tanabe M., Sato Y., Morishima K. (2017). KEGG: New perspectives on genomes, pathways, diseases and drugs. Nucleic Acids Res..

[43] Jensen L.J., Julien P., Kuhn M., von Mering C., Muller J., Doerks T., Bork P. (2007). EggNOG: Automated construction and annotation of orthologous groups of genes. Nucleic Acids Res..

[44] Brown C.T., Hug L.A., Thomas B.C., Sharon I., Castelle C.J., Singh A., Wilkins M.J., Wrighton K.C., Williams K.H., Banfield J.F. (2015). Unusual biology across a group comprising more than 15% of domain bacteria. Nature.

[45] Frey B., Rime T., Phillips M., Stierli B., Hajdas I., Widmer F., Hartmann M. (2016). Microbial diversity in European alpine permafrost and active layers. FEMS Microbiol. Ecol..

[46] Herrmann M., Wegner C.-E., Taubert M., Geesink P., Lehmann K., Yan L., Lehmann R., Totsche K.U., Küsel K. (2019). Predominance of cand. patescibacteria in groundwater is caused by their preferential mobilization from soils and flourishing under oligotrophic conditions. Front. Microbiol..

[47] Hubalek V., Wu X., Eiler A., Buck M., Heim C., Dopson M., Bertilsson S., Ionescu D. (2016). Connectivity to the surface determines diversity patterns in subsurface aquifers of the Fennoscandian shield. ISME J..

[48] León-Zayas R., Peoples L., Biddle J.F., Podell S., Novotny M., Cameron J., Lasken R.S., Bartlett D.H. (2017). The metabolic potential of the single cell genomes obtained from the Challenger Deep, Mariana Trench within the candidate superphylum P arcubacteria (OD 1). Environ. Microbiol..

[49] Luef B., Frischkorn K.R., Wrighton K.C., Holman H.-Y.N., Birarda G., Thomas B.C., Singh A., Williams K.H., Siegerist C.E., Tringe S.G. (2015). Diverse uncultivated ultra-small bacterial cells in groundwater. Nat. Commun..

[50] Antunes T.C., Marconatto L., Borges L.G.d.A., Giongo A., Sand S.T.V.D. (2021). Analysis of microbial community biodiversity in activated sludge from a petrochemical plant. Rev. Ambiente Água.

[51] Eze M.O., Lütgert S.A., Neubauer H., Balouri A., Kraft A.A., Sieven A., Daniel R., Wemheuer B. (2020). Metagenome assembly and metagenome-assembled genome sequences from a historical oil field located in Wietze, Germany. Microbiol. Resour. Announc..

[52] Tian R., Ning D., He Z., Zhang P., Spencer S.J., Gao S., Shi W., Wu L., Zhang Y., Yang Y. (2020). Small and mighty: Adaptation of superphylum Patescibacteria to groundwater environment drives their genome simplicity. Microbiome.

[53] Castelle C.J., Brown C.T., Anantharaman K., Probst A.J., Huang R.H., Banfield J.F. (2018). Biosynthetic capacity, metabolic variety and unusual biology in the CPR and DPANN radiations. Nat. Rev. Microbiol..

[54] Shelton J.L., Andrews R.S., Akob D.M., DeVera C.A., Mumford A., McCray J.E., McIntosh J.C. (2018). Microbial community composition of a hydrocarbon reservoir 40 years after a CO2 enhanced oil recovery flood. FEMS Microbiol. Ecol..

[55] Alsouleman K., Linke B., Klang J., Klocke M., Krakat N., Theuerl S. (2016). Reorganisation of a mesophilic biogas microbiome as response to a stepwise increase of ammonium nitrogen induced by poultry manure supply. Bioresour. Technol..

[56] Gao P., Tian H., Wang Y., Li Y., Li Y., Xie J., Zeng B., Zhou J., Li G., Ma T. (2016). Spatial isolation and environmental factors drive distinct bacterial and archaeal communities in different types of petroleum reservoirs in China. Sci. Rep..

[57] Zhao J.-Y., Hu B., Dolfing J., Li Y., Tang Y.-Q., Jiang Y., Chi C.-Q., Xing J., Nie Y., Wu X.-L. (2021). Thermodynamically favorable reactions shape the archaeal community affecting bacterial community assembly in oil reservoirs. Sci. Total Environ..

[58] Wang H.-Z., Gou M., Yi Y., Xia Z.-Y., Tang Y.-Q. (2018). Identification of novel potential acetate-oxidizing bacteria in an acetate-fed methanogenic chemostat based on DNA stable isotope probing. J. Gen. Appl. Microbiol..

[59] Sengupta K., Pal S. (2021). A review on microbial diversity and genetic markers involved in methanogenic degradation of hydrocarbons: Futuristic prospects of biofuel recovery from contaminated regions. Environ. Sci. Pollut. Res..

[60] Oren A. (2014). The family Methanotrichaceae. The Prokaryotes.

[61] Akinyemi T.S., Shao N., Whitman W.B. (2014). Methanothrix. Bergey’s Manual of Systematics of Archaea and Bacteria.

[62] Holmes D.E., Shrestha P.M., Walker D.J.F., Dang Y., Nevin K.P., Woodard T.L., Lovley D.R. (2017). Metatranscriptomic evidence for direct interspecies electron transfer between geobacter and methanothrix species in methanogenic rice paddy soils. Appl. Environ. Microbiol..

[63] John E.S., Flores G.E., Meneghin J., Reysenbach A.-L. (2019). Deep-sea hydrothermal vent metagenome–Assembled genomes provide insight into the phylum Nanoarchaeota. Environ. Microbiol. Rep..

[64] Munson-McGee J.H., Field E.K., Bateson M., Rooney C., Stepanauskas R., Young M.J., Wommack K.E. (2015). Nanoarchaeota, their sulfolobales host, and nanoarchaeota virus distribution across Yellowstone national park hot springs. Appl. Environ. Microbiol..

[65] Carrier V., Svenning M.M., Gründger F., Niemann H., Dessandier P.-A., Panieri G., Kalenitchenko D. (2020). The impact of methane on microbial communities at marine arctic gas hydrate bearing sediment. Front. Microbiol..

[66] Casanueva A., Galada N., Baker G.C., Grant W.D., Heaphy S., Jones B., Yanhe M., Ventosa A., Blamey J., Cowan D.A. (2008). Nanoarchaeal 16S rRNA gene sequences are widely dispersed in hyperthermophilic and mesophilic halophilic environments. Extremophiles.

[67] Chernyh N.A., Mardanov A.V., Gumerov V.M., Miroshnichenko M.L., Lebedinsky A.V., Merkel A.Y., Crowe D., Pimenov N.V., Rusanov I.I., Ravin N.V. (2015). Microbial life in Bourlyashchy, the hottest thermal pool of Uzon Caldera, Kamchatka. Extremophiles.

[68] Clingenpeel S., Kan J., Macur R., Woyke T., Lovalvo D., Varley J., Inskeep W.P., Nealson K., McDermott T. (2013). Yellowstone lake nanoarchaeota. Front. Microbiol..

[69] Flores G., Shakya M., Meneghin J., Yang Z., Seewald J., Geoff Wheat C., Podar M., Reysenbach A.L. (2012). Inter-field variability in the microbial communities of hydrothermal vent deposits from a back-arc basin. Geobiology.

[70] Flores G.E., Campbell J.H., Kirshtein J.D., Meneghin J., Podar M., Steinberg J.I., Seewald J.S., Tivey M.K., Voytek M.A., Yang Z.K. (2011). Microbial community structure of hydrothermal deposits from geochemically different vent fields along the Mid-Atlantic Ridge. Environ. Microbiol..

[71] Hohn M.J., Hedlund B.P., Huber H. (2002). Detection of 16S rDNA sequences representing the novel phylum “Nanoarchaeota”: Indication for a wide distribution in high temperature biotopes. Syst. Appl. Microbiol..

[72] McCliment E.A., Voglesonger K.M., O’Day P.A., Dunn E.E., Holloway J.R., Cary S.C. (2006). Colonization of nascent, deep-sea hydrothermal vents by a novel Archaeal and Nanoarchaeal assemblage. Environ. Microbiol..

[73] Zemskaya T.I., Cabello-Yeves P.J., Pavlova O.N., Rodriguez-Valera F. (2020). Microorganisms of lake Baikal—The deepest and most ancient lake on Earth. Appl. Microbiol. Biotechnol..

[74] Mayumi D., Mochimaru H., Tamaki H., Yamamoto K., Yoshioka H., Suzuki Y., Kamagata Y., Sakata S. (2016). Methane production from coal by a single methanogen. Science.

[75] Cheng L., Ding C., Li Q., He Q., Dai L.-R., Zhang H. (2013). DNA-SIP reveals that syntrophaceae play an important role in methanogenic hexadecane degradation. PLoS ONE.

[76] Gao P., Tian H., Li G., Sun H., Ma T. (2015). Microbial diversity and abundance in the Xinjiang Luliang long-term water-flooding petroleum reservoir. Microbiol. Open.

[77] Walker D.J.F., Adhikari R.Y., Holmes D.E., Ward J.E., Woodard T.L., Nevin K.P., Lovley D.R. (2018). Electrically conductive pili from pilin genes of phylogenetically diverse microorganisms. ISME J..

[78] Greene A.C., Patel B.K.C., Yacob S. (2009). Geoalkalibacter subterraneus sp. nov., an anaerobic Fe(III)- and Mn(IV)-reducing bacterium from a petroleum reservoir, and emended descriptions of the family Desulfuromonadaceae and the genus Geoalkalibacter. Int. J. Syst. Evol. Microbiol..

[79] Piceno Y.M., Reid F.C., Tom L.M., Conrad M.E., Bill M., Hubbard C.G., Fouke B.W., Graff C.J., Han J., Stringfellow W.T. (2014). Temperature and injection water source influence microbial community structure in four Alaskan North Slope hydrocarbon reservoirs. Front. Microbiol..

[80] Mayilraj S., Kaksonen A.H., Cord-Ruwisch R., Schumann P., Spröer C., Tindall B.J., Spring S. (2009). Desulfonauticus autotrophicus sp. nov., a novel thermophilic sulfate-reducing bacterium isolated from oil-production water and emended description of the genus Desulfonauticus. Extremophiles.

[81] Orphan V., Taylor L., Hafenbradl D., Delong E. (2000). Culture-dependent and culture-independent characterization of microbial assemblages associated with high-temperature petroleum reservoirs. Appl. Environ. Microbiol..

[82] Shestakova N., Ivoilov V., Tourova T., Belyaev S., Poltaraus A., Nazina T. (2011). Application of clone libraries: Syntrophic acetate degradation to methane in a high-temperature petroleum reservoir: Culture-based and 16S rRNA genes characterization. Applied Microbiology and Molecular Biology in Oil Field Systems.

[83] Mnif S., Chamkha M., Sayadi S. (2009). Isolation and characterization of Halomonas sp. strain C2SS100, a hydrocarbon-degrading bacterium under hypersaline conditions. J. Appl. Microbiol..

[84] Cheng L., He Q., Ding C., Dai L.-R., Li Q., Zhang H. (2013). Novel bacterial groups dominate in a thermophilic methanogenic hexadecane-degrading consortium. FEMS Microbiol. Ecol..

[85] Mbadinga S.M., Li K.-P., Zhou L., Wang L.-Y., Yang S.-Z., Liu J.-F., Gu J.-D., Mu B.-Z. (2012). Analysis of alkane-dependent methanogenic community derived from production water of a high-temperature petroleum reservoir. Appl. Microbiol. Biotechnol..

[86] Zhou L., Li K.-P., Mbadinga S.M., Yang S.-Z., Gu J.-D., Mu B.-Z. (2012). Analyses of n-alkanes degrading community dynamics of a high-temperature methanogenic consortium enriched from production water of a petroleum reservoir by a combination of molecular techniques. Ecotoxicology.

[87] Pournia M., Bahador N., Tabatabaei M., Azarbayjani R., Salekdeh G.H. (2020). Microbial diversity of non-flooded high temperature petroleum reservoir in South of Iran. Biol. J. Microorg..

[88] Dahle H., Garshol F., Madsen M., Birkeland N.-K. (2008). Microbial community structure analysis of produced water from a high-temperature North Sea oil-field. Antonie Van Leeuwenhoek.

[89] Magot M., Ollivier B., Patel B.K.C. (2000). Microbiology of petroleum reservoirs. Antonie Van Leeuwenhoek.

[90] Henry E.A., Devereux R., Maki J.S., Gilmour C.C., Woese C.R., Mandelco L., Schauder R., Remsen C.C., Mitchell R. (1994). Characterization of a new thermophilic sulfate-reducing bacterium. Arch. Microbiol..

[91] Meslé M., Dromart G., Oger P. (2013). Microbial methanogenesis in subsurface oil and coal. Res. Microbiol..

[92] Nazina T.N., Shestakova N.M., Semenova E.M., Korshunova A.V., Kostrukova N.K., Tourova T.P., Min L., Feng Q., Poltaraus A.B. (2017). Diversity of metabolically active bacteria in water-flooded high-temperature heavy oil reservoir. Front. Microbiol..

[93] Silva T.R., Verde L.C.L., Santos Neto E.V., Oliveira V.M. (2013). Diversity analyses of microbial communities in petroleum samples from Brazilian oil fields. Int. Biodeterior. Biodegrad..

[94] Liu J.-F., Wu W.-L., Yao F., Wang B., Zhang B.-L., Mbadinga S.M., Gu J.-D., Mu B.-Z. (2016). A thermophilic nitrate-reducing bacterium isolated from production water of a high temperature oil reservoir and its inhibition on sulfate-reducing bacteria. Appl. Environ. Biotechnol..

[95] Tüccar T., Ilhan-Sungur E., Abbas B., Muyzer G. (2019). Coexistence of sulfate reducers with the other oil bacterial groups in Diyarbakır oil fields. Anaerobe.

[96] Nazaries L., Murrell J.C., Millard P., Baggs L., Singh B.K. (2013). Methane, microbes and models: Fundamental understanding of the soil methane cycle for future predictions. Environ. Microbiol..

[97] Penger J., Conrad R., Blaser M. (2012). Stable carbon isotope fractionation by methylotrophic methanogenic archaea. Appl. Environ. Microbiol..

[98] Santos J.C.D., Lopes D.R.G., Da Silva J.D., De Oliveira M.D., Dias R.S., Lima H.S., De Sousa M.P., De Paula S.O., Silva C.C.D. (2020). Diversity of sulfate-reducing prokaryotes in petroleum production water and oil samples. Int. Biodeterior. Biodegrad..

[99] Summers Z.M., Belahbib H., Pradel N., Bartoli M., Mishra P., Tamburini C., Dolla A., Ollivier B., Armougom F. (2020). A novel Thermotoga strain TFO isolated from a Californian petroleum reservoir phylogenetically related to Thermotoga petrophila and T. naphthophila, two thermophilic anaerobic isolates from a Japanese reservoir: Taxonomic and genomic considerations. Syst. Appl. Microbiol..

[100] Gieg L. (2018). Microbial communities in oil shales, biodegraded and heavy oil reservoirs, and bitumen deposits. Microbial Communities Utilizing Hydrocarbons and Lipids: Members, Metagenomics and Ecophysiology.

[101] Qiu Y.-L., Hanada S., Ohashi A., Harada H., Kamagata Y., Sekiguchi Y. (2008). Syntrophorhabdus aromaticivorans gen. nov., sp. nov., the first cultured anaerobe capable of degrading phenol to acetate in obligate syntrophic associations with a hydrogenotrophic methanogen. Appl. Environ. Microbiol..

[102] Kuever J., Rosenberg E., DeLong E.F., Lory S., Stackebrandt E., Thompson F. (2014). The family Syntrophorhabdaceae. The Prokaryotes: Deltaproteobacteria and Epsilonproteobacteria.

[103] Dong X., Greening C., Rattray J.E., Chakraborty A., Chuvochina M., Mayumi D., Dolfing J., Li C., Brooks J.M., Bernard B.B. (2019). Metabolic potential of uncultured bacteria and archaea associated with petroleum seepage in deep-sea sediments. Nat. Commun..

[104] Aitken C.M., Jones D.M., Maguire M.J., Gray N.D., Sherry A., Bowler B.F.J., Ditchfield A.K., Larter S.R., Head I.M. (2013). Evidence that crude oil alkane activation proceeds by different mechanisms under sulfate-reducing and methanogenic conditions. Geochim. Cosmochim. Acta.

[105] Davis J.J., Wattam A.R., Aziz R.K., Brettin T., Butler R., Butler R.M., Chlenski P., Conrad N., Dickerman A., Dietrich E.M. (2020). The PATRIC bioinformatics resource center: Expanding data and analysis capabilities. Nucleic Acids Res..

[106] Wang S.C. (2003). Studies of Bacterial Catabolic Enzymes: Implications for the Evolution of Enzymes and Metabolic Pathways. Ph.D. Thesis.

[107] Davidson R., Baas B.-J., Akiva E., Holliday G.L., Polacco B.J., LeVieux J.A., Pullara C.R., Zhang Y.J., Whitman C.P., Babbitt P.C. (2018). A global view of structure–function relationships in the tautomerase superfamily. J. Biol. Chem..

[108] Michał B., Gagat P., Jabłoński S., Chilimoniuk J., Gaworski M., Mackiewicz P., Marcin Ł., Burdukiewicz M., Łukaszewicz M. (2018). PhyMet2: A database and toolkit for phylogenetic and metabolic analyses of methanogens. Environ. Microbiol. Rep..

[109] Borrel G., Adam P.S., McKay L.J., Chen L.-X., Sierra-García I.N., Sieber C.M.K., Letourneur Q., Ghozlane A., Andersen G.L., Li W.-J. (2019). Wide diversity of methane and short-chain alkane metabolisms in uncultured archaea. Nat. Microbiol..

[110] Laso-Pérez R., Hahn C., Vliet D.M.V., Tegetmeyer H.E., Schubotz F., Smit N.T., Pape T., Sahling H., Bohrmann G., Boetius A. (2019). Anaerobic degradation of non-methane alkanes by candidatus methanoliparia in hydrocarbon seeps of the gulf of Mexico. mBio.

[111] Lahme S., Callbeck C.M., Eland L.E., Wipat A., Enning D., Head I.M., Hubert C.R.J. (2020). Comparison of sulfide-oxidizing Sulfurimonas strains reveals a new mode of thiosulfate formation in subsurface environments. Environ. Microbiol..

[112] Dahle H., Roalkvam I., Thorseth I.H., Pedersen R.B., Steen I.H. (2013). The versatile in situ gene expression of an E psilonproteobacteria-dominated biofilm from a hydrothermal chimney. Environ. Microbiol. Rep..

[113] Hensen D., Sperling D., Trüper H.G., Brune D.C., Dahl C. (2006). Thiosulphate oxidation in the phototrophic sulphur bacterium Allochromatium vinosum. Mol. Microbiol..

[114] Zumft W., Kroneck P., Poole R.K. (2007). Respiratory transformation of nitrous oxide (N_2_O) to dinitrogen by bacteria and archaea. Advances in Microbial Physiology.

