# Supplementary figures and images for: Genome-Resolved Meta-Analysis of the Microbiome in Oil Reservoirs Worldwide
Source: Microorganisms. 2021 Aug 26;9(9):1812. doi: 10.3390/microorganisms9091812 (PMC8465018; doi:10.3390/microorganisms9091812)

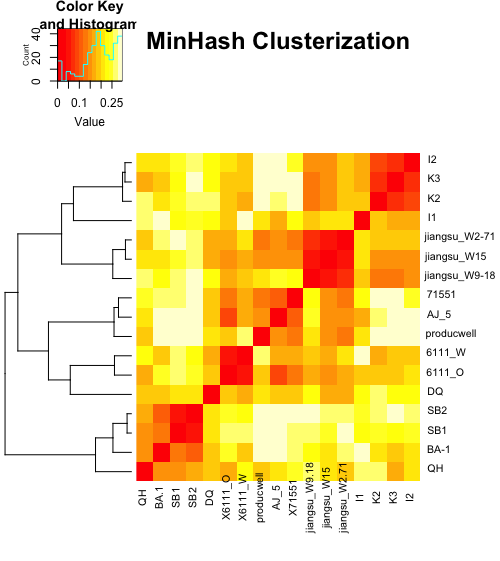

Supplement: Supplementary file 1 [file microorganisms-09-01812-s001.zip › FIGS1.png]

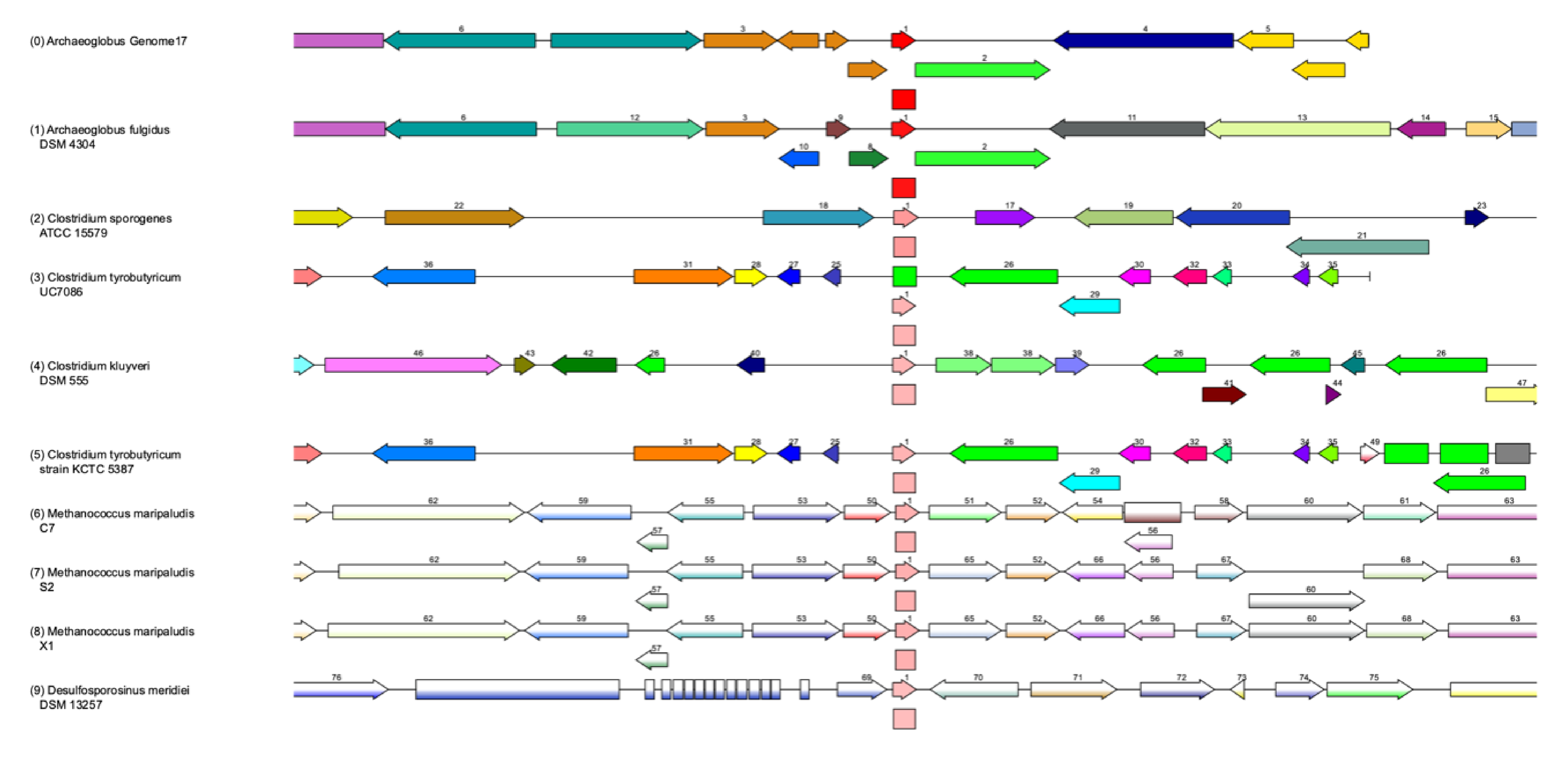

Supplement: Supplementary file 1 [file microorganisms-09-01812-s001.zip › FIGS3a.png]

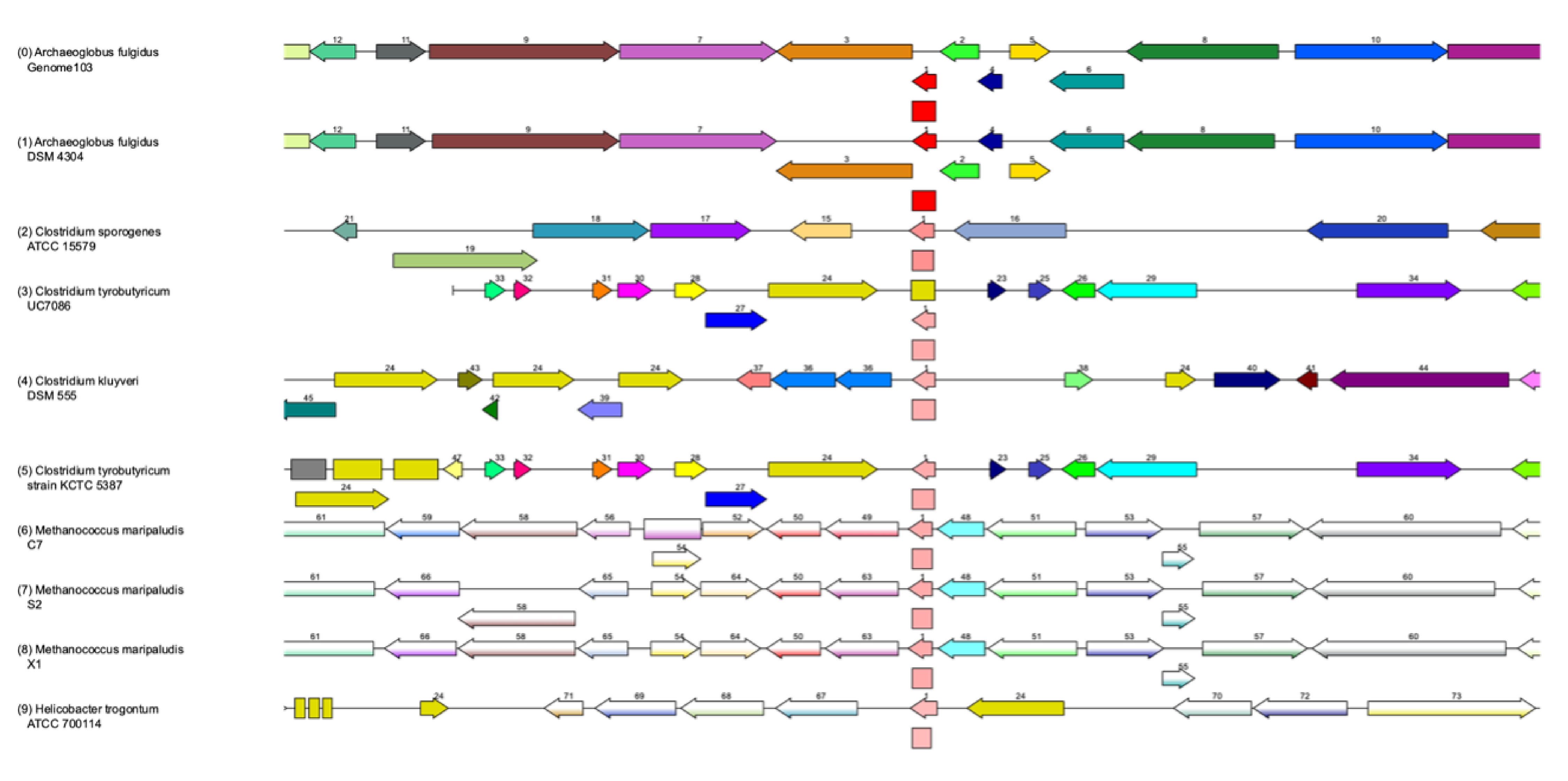

Supplement: Supplementary file 1 [file microorganisms-09-01812-s001.zip › FIGS3b.png]

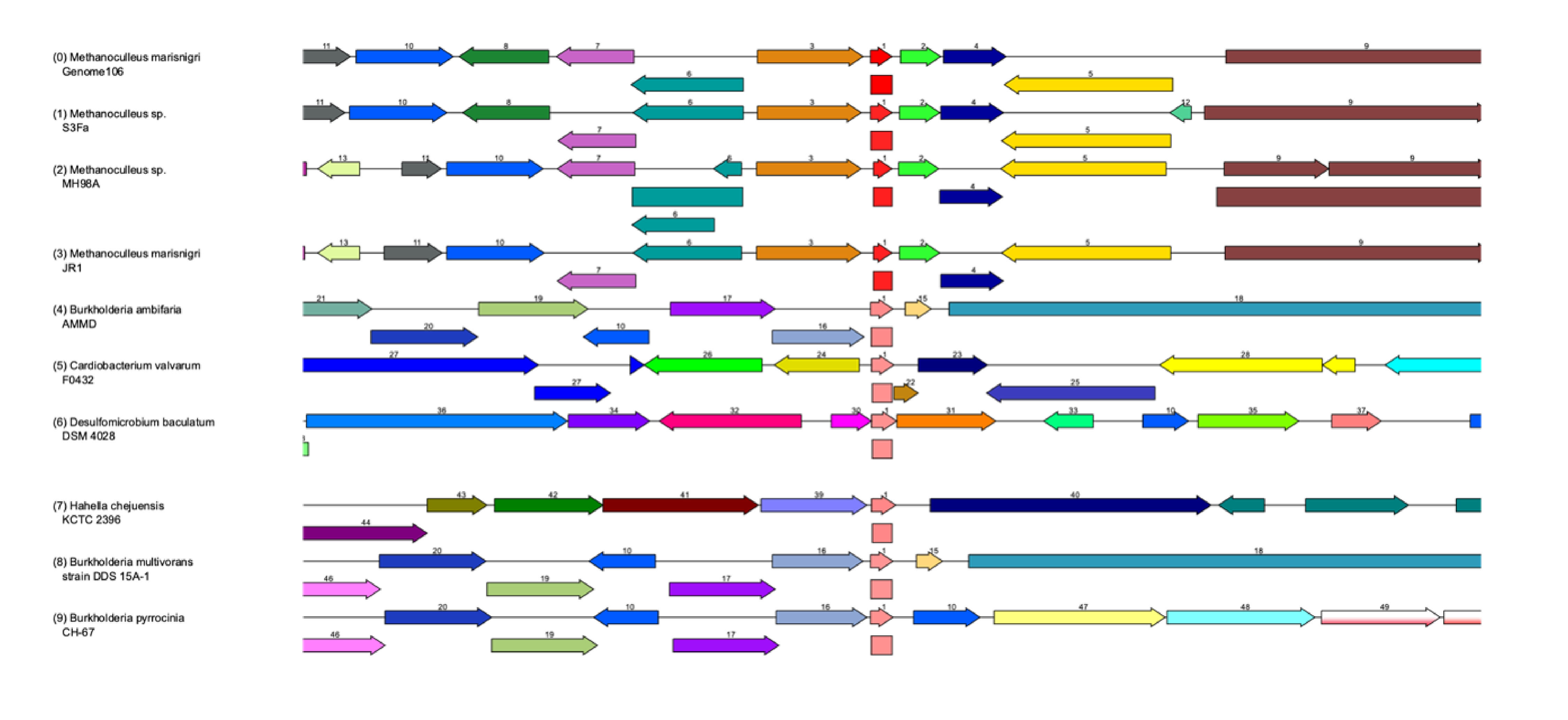

Supplement: Supplementary file 1 [file microorganisms-09-01812-s001.zip › FIGS3c.png]
